# Maternal Prenatal Cortisol and Breastfeeding Predict Infant Growth

**DOI:** 10.3390/ijerph17218233

**Published:** 2020-11-07

**Authors:** Nicki L. Aubuchon-Endsley, Hillary E. Swann-Thomsen, Nicole Douthit

**Affiliations:** 1 Department of Psychology, Idaho State University, Pocatello, ID 83209, USA; 2 Idaho Center for Health Research, Idaho State University, Meridian, ID 83642, USA; swanhill@isu.edu; 3 Frenova Renal Research, Meridian, ID 83642, USA; doutnico@isu.edu

**Keywords:** maternal, prenatal, cortisol, breastfeeding, infant growth

## Abstract

Fetal/infant growth affects adult obesity and morbidities/mortality and has been associated with prenatal exposure to cortisol. Bidirectional relations between maternal stress and breastfeeding suggest that they interact to influence offspring growth. No models have tested this hypothesis, particularly regarding longer-term offspring outcomes. We used a subset of the IDAHO Mom Study (*n* = 19–95) to examine associations among maternal prenatal cortisol (cortisol awakening response (CAR) and area under the curve), and standardized weight-for-length (WLZ) and length-for-age (LAZ) z-scores from birth-18 months, and main and interactive effects of prenatal cortisol and breastfeeding on infant growth from birth-6 months. CAR was negatively associated with LAZ at birth (*r* = −0.247, *p* = 0.039) but positively associated at 13–14 months (*r* = 0.378, *p* = 0.033), suggesting infant catch-up growth with lower birth weights, likely related to elevated cortisol exposure, continues beyond early infancy. A negative correlation between breastfeeding and 10-month WLZ (*r* = −0.344, *p* = 0.037) and LAZ (*r* = −0.468, *p* = 0.005) suggests that breastfeeding assists in managing infant growth. WLZ and LAZ increased from birth to 6 months (*p*s < 0.01), though this was unrelated to interactions between prenatal cortisol and breastfeeding (i.e., no significant moderation), suggesting that other factors played a role, which should be further investigated. Findings add to our understanding of the predictors of infant growth.

## 1. Introduction

Fetal and infant growth influence the risk of obesity, morbidity, (e.g., diabetes mellitus, cardiovascular disease, hypertension, sleep apnea, kidney and liver disease, infertility, gastroesophageal reflux disease, and certain cancers), and mortality across the lifespan [1]. However, early biopsychosocial risk factors influencing fetal/infant growth are still not well understood. Therefore, additional research is needed to investigate maternal perinatal factors, which influence early offspring growth to identify early targets for obesity prevention and intervention. While literature supports two such factors, in utero glucocorticoid exposure and breastfeeding, no known studies have investigated an interaction between these factors in relation to offspring growth trajectories over infancy, which is the focus of the current project.

### 1.1. Cortisol

During pregnancy, maternal cortisol is regulated by the placenta, uterine decidua, and fetal membrane, in addition to the hypothalamic–pituitary–adrenal (HPA) axis [2]. Significant increases in cortisol occur during pregnancy, classifying it as a physiologic period of hypercortisolism; however, notably, the variation of the diurnal rhythm of cortisol remains fairly consistent during pregnancy [3], though there may be seasonal effects [4]. Due to transfer through the bloodstream of the mother and infant, as well as placental transfer, increased maternal cortisol levels result in increased cortisol exposure to the fetus [2]. Research in human and non-human animal studies have suggested that exposure to glucocorticoids during prenatal development is necessary for typical development, however, continued exposure to high levels of cortisol can have adverse impacts on development and growth, including preterm delivery, intrauterine growth restriction, and low birth weight [5,6,7].

Moreover, there are several indices of cortisol that have been validated in human research with perhaps the most common including area under the curve (AUC) and cortisol awakening response (CAR) [8]. The area under the curve with respect to ground (AUCg) takes into account change over time and the distance of each cortisol measurement from zero and is a valid estimate of total daily cortisol secretion [8]. CAR is the unique spike in cortisol (about 50% increase) that occurs when waking in the morning and reflects a distinct aspect of HPA axis activity that helps to more comprehensively understand the diurnal cortisol curve. In particular, it is postulated that it may be an index of adrenal capacity to respond to stress and awakening [8]. Therefore, both indices were examined in the current study.

### 1.2. Maternal Cortisol and Offspring Growth

The impact of maternal prenatal cortisol and resulting low birth weight has important implications for offspring outcomes, including increased risk of cardiovascular and metabolic disorders (e.g., hypertension, coronary heart disease, type II diabetes mellitus, insulin resistance, and hyperlipidemia), depression, schizophrenia, and autism [2]. Research suggests that these health conditions may be related to early growth patterns. For example, rapid growth, often referred to as catch-up growth, occurs as a result of preterm birth and low birth weight—potential consequences of elevated maternal cortisol exposure [9]. In adults who were born preterm or had low birth weight, and likely experienced rapid catch-up growth, there was an increased risk of developing a cardiovascular or metabolic disorder (e.g., type II diabetes mellitus, heart disease, or stroke), as well as increased risk of obesity [10,11].

### 1.3. Breastfeeding

Further impacting infant growth is postnatal infant feeding practices. Whether or not an infant is breastfed, as well as the duration of breastfeeding, has been found to be associated with infant growth. Previous research has found that infants who were not breastfed had, on average, a lower weight compared to infants who were breastfed until 4 to 6 months of age [11,12]. Similarly, infants younger than 6 months who were not breastfed had lower weight-for-length z-scores (WLZ) compared to infants who were breastfed [12]. However, at around 6 months of age, infants who were not breastfed demonstrated rapid catch-up growth [11]. Again, this rapid catch-up growth, whether as a result of elevated maternal prenatal cortisol or differences in infant feeding practices, is associated with adverse outcomes in offspring including chronic diseases later in life [12]. Additionally, the rates of chronic diseases such as allergies, obesity, diabetes mellitus, hypertension, Crohn’s disease, and cancer are lower in adults who were breastfed as infants [11].

### 1.4. Summary

Elevated cortisol levels are known to lead to stunted prenatal growth [5] and decreased birth weight, which in turn leads to rapid catch-up growth and steeper infant growth trajectories, depending on the infant’s diet [12]. Specifically, breastfeeding assists in promoting healthy infant weight and growth and breastfed babies are less likely to have issues with obesity in childhood and adulthood [11]. Moreover, excessive maternal stress may lead to lactation insufficiency, so it is possible that elevated maternal cortisol levels will be associated with a decreased ability for mothers to breastfeed, thus further interrupting healthy infant growth patterns [13]. However, the relations between fetal cortisol exposure, longer-term infant growth outcomes, and breastfeeding together are unknown. Given the role that each of these factors play, it is important to examine their unique and combined effects, to further elucidate how an infant’s growth, development, and future health outcomes may be impacted.

### 1.5. Hypotheses

It is hypothesized that there will be significant negative relations between maternal prenatal diurnal cortisol rhythms (i.e., area-under-the-curve with respect to ground (AUC_G_) and cortisol awakening response (CAR)) and infant standardized anthropometric growth outcomes (i.e., weight-for-length (WLZ) and length-for-age z-scores (LAZ)) and that less breastfeeding at 6 months will increase the strength of this relationship. Specifically, we hypothesize that: (1) greater prenatal maternal circadian cortisol release (i.e., AUC_G_ and CAR) will be related to lower infant WLZ (a) and LAZ (b) from birth to 6 months and (2) the negative relationships between greater maternal prenatal cortisol (i.e., AUC_G_ and CAR) and lower infant WLZ (a) and LAZ (b) at 6 months will be exacerbated by less breastfeeding (i.e., lower frequency and shorter duration) at 6 months.

## 2. Materials and Methods

### 2.1. Recruitment and Screening

All procedures and materials for the current project were approved by the Idaho State University (Pocatello, ID, USA) Human Subjects Committee. Data for this project were drawn from the larger, longitudinal Infant Development and Healthy Outcomes in Mothers (IDAHO Mom) Study, which examined mothers’ health and behavior during pregnancy and infants’ growth and development at 6, 10, 13–14, and 18 months postpartum. Participants were recruited from local businesses, schools, and doctor’s offices with flyers, brochures, and in-person educational sessions, as well as advertisements on social media platforms and local newspapers, radio, and television stations. Potential participants were contacted and briefly screened prior to being invited to participate in the study. Following oral consent for screening, potential participants were queried on the following exclusion criteria: younger than 18 or older than 35 years of age, consumption of United States Food and Drug Administration (FDA) Category D or X medications during pregnancy (i.e., the FDA provides safety and regulatory information for drugs in reference to fetal and maternal risk during pregnancy to consumers, providers, and industry), being pregnant with multiple fetuses, having a diagnosis of a severe and persistent mental illness (e.g., a bipolar or psychotic spectrum disorder), the anticipated involvement of Department of Health and Welfare after birth, being over 37 weeks gestation, and having a major medical condition (e.g., gestational diabetes, pre-eclampsia, toxemia, or HIV/AIDS) that would result in being considered a high-risk pregnancy. Eligible participants were scheduled for a prenatal session between 33 and 37 weeks gestation.

### 2.2. Prenatal Session and Salivary Cortisol Samples

The prenatal session consisted of trained research assistants (at least one undergraduate and one graduate) providing instructions and support to participants during written informed consent procedures, interviews, self-report questionnaires including demographic information, such as ethnicity, household income, and education level, and measurements of their height, weight, and waist circumference as part of the larger study. Participants were compensated for their participation with a monetary award of $30 per session. Following the completion of the laboratory session, participants were provided with a saliva collection kit to measure diurnal cortisol for a 3-day collection period, collecting four samples per day, including: immediately after wakening, 30 min post-awakening, 45 min post-awakening, and prior to going to bed at night. The passive drool method was used for saliva collection because it provides the purest sample possible of whole saliva (SalivaBio Passive Drool). Participants recorded their saliva collection times on the instruction sheet and also sent a text message to the IDAHO Mom Study cellular phone and linked e-mail account when they collected each sample if they opted to receive automatic text message reminders. They were instructed not to eat, drink, smoke, or brush their teeth prior to collecting the samples and then to place each sample in their freezer after they were collected. Participants were compensated $5 for each day of completed saliva collection. A total of 30 out of the 125 mothers in the larger IDAHO Mom Study did not return saliva kits. Once the saliva collection kit was returned to the laboratory, saliva specimens were stored in a −20 °C freezer until sent to the ISU Molecular Research Core Facility (MRCF) for cortisol assay (ELISA, Salimetrics). The sensitivity of the Salimetrics kits was <0.007 ug/dL and the assay range for the kits was 0.012–3.00 ug/dL (SalivaBio Passive Drool).

Once the ELISA assays were completed, a coefficient of variation (CV%) was calculated to determine the intra-replicate variation. Sample concentrations were eliminated if the CV% between the replicates exceeded 15% and the concentration values differed by 0.007 ug/dL. Cortisol concentrations greater than 4 ug/dL were eliminated, as they are unlikely to be physiologically plausible. From the cortisol concentration values, the cortisol awakening response (CAR) and area under the curve with respect to ground (AUC_G_) were calculated and used for statistical analyses. CAR is calculated by computing a change score between cortisol concentration at awakening and cortisol concentration post-awakening [14]. AUC_G_ is calculated by summing a series of trapezoids that reflected repeated measurements of cortisol [14]. Values from the 3-day sampling were averaged to create mean CAR and AUC_G_ values for each participant. AUC indicates the average amount of circulating cortisol, while CAR represents the morning spike in circulating cortisol.

### 2.3. Postnatal Sessions

Participants were asked to return to the lab for four follow-up sessions when their infant was 6, 10, 13–14, and 18 months old (±2 weeks). Anthropometric data from mothers and infants were collected at each session. Infant birth weight and length were reported via a maternal report at the 6-month session. Infant length was measured using a ShorrBoard (±0.1 cm), infant weight was assessed using a Seca mother–infant scale (±100 g), and infant waist circumference was measured with a ShorrTape (±0.1 cm). The scale was calibrated monthly to ensure accurate measurements. Infant weight was measured once, whereas length and waist circumference were collected twice and averaged across the two measurements. Measurements were collected in kilogram and centimeter units. Z-scores for Weight-for-Length (WLZ) and Length-for-Age (LAZ) were calculated based upon infants’ sex and age at birth and each follow-up session via conversion software utilizing the WHO Child Growth Standards (version 3.2.2, January 2011) [15]. To calculate the frequency and duration of breastfeeding at each follow-up session, mothers completed the Infant Dietary Questionnaire (IDQ) [16]. The questionnaire item that was used for breastfeeding frequency data analyses was “Presently, for how many feedings a day do you breastfeed?” The questionnaire items that were used for breastfeeding duration data analyses were “Are you currently breastfeeding your baby?” and “If no, for how long did you breastfeed your baby?” Participants indicated length in months, weeks, and/or days. The items on this questionnaire are based on the CDC’s Infant Feeding Practices Study II [16] and are not intended to measure breastfeeding exclusivity (i.e., mothers could have been supplementing with formula or bottle feeding with breastmilk).

### 2.4. Statistical Analyses

Sample sizes varied across analyses following data cleaning and therefore are reported in the respective analyses sections. IBM SPSS Statistics (version 24.0) was used for all analyses. Descriptive statistics for demographic variables were conducted to characterize the sample. In order to better understand the relationship between maternal prenatal circadian cortisol release and infant growth, a series of correlations were run to probe the relationship between WLZ and LAZ at birth, 6 months, 10 months, 13–14 months, and 18 months and the cortisol indices (AUC_G_ and CAR). Correlation analyses were also conducted to examine associations among breastfeeding duration and frequency and infant growth from birth to 18 months. Next, a series of two-way Mixed Analyses of Variance (Mixed ANOVAs) were conducted to test relations between maternal prenatal circadian cortisol release and infant growth from birth to 6 months. Time served as the within-subjects independent variable, maternal prenatal diurnal cortisol indices served as the between-subjects independent variables, and infant anthropometric variables served as dependent variables, in separate models.

Moderation analyses were conducted using Hayes’ PROCESS macro (version 3.1) to determine whether maternal breastfeeding frequency and duration moderate the relationship between maternal prenatal diurnal cortisol concentrations and infant growth. A moderation model was conducted for each outcome variable, Y, including WLZ and LAZ, at 6 months with separate models for each primary predictor variable, X, including AUC_G_ and CAR, and the moderator variable, W, was either breastfeeding frequency or breastfeeding duration, again in separate models. Potential covariates, including gestational age, preterm birth, low birth weight, pre-pregnancy weight, gestational weight gain, diet, and gravida, were examined, but were not found to be predictive of the outcome variables in this sample and thus were not included in the models.

## 3. Results

### 3.1. Demographic Information

Participants were primarily European-American (93%) with an average age of 26.84 (±4.39, *N* = 125) years at the time of enrollment in the study. Seventy-nine percent of participants indicating that they were married, 10% reported being in a committed relationship or engaged, 8% reported being single and 2% indicated that they were divorced. Approximately 54% of participants would be considered middle class, making $30,000 to $100,000 annually with the majority (82%) having continued education beyond high school (see Table 1). The average gestational age at birth was 39.5 weeks. The average infant age was 6.1 months at the 6-month session, 10.1 months at the 10-month session, 14.1 months at the 13–14-month session, and 18.1 months at the 18-month session. Approximately 47% of infants were female and 53% were male.

### 3.2. Correlations

Pearson’s product–moment correlations were conducted to assess relations among infant growth indices, including WLZ and LAZ, from birth to 18 months and maternal prenatal cortisol indices, including AUC_G_ and CAR (See Table 1). There was a significant negative correlation between infant LAZ at birth and maternal prenatal CAR (*r*(70) = −0.25, *p* < 0.05) and a significant positive correlation between infant LAZ at 13–14 months and maternal prenatal CAR (*r*(32) = 0.38, *p* < 0.05). There were no statistically significant correlations between infant LAZ at 10 or 18 months and maternal prenatal cortisol. Additionally, there were no statistically significant correlations between infant WLZ and maternal prenatal cortisol indices at any time point.

Pearson’s product–moment correlations were completed to examine associations among breastfeeding and infant growth indices from birth to 18 months (See Table 2). There was a significant negative correlation between breastfeeding frequency (range = 0–12 feedings per day) and infant LAZ at 10 months (*r*(35) = −0.47, *p* < 0.005) and breastfeeding duration (range = 0–180 days of breastfeeding through 6 months) and infant WLZ at 10 months (*r*(37) = −0.34, *p* = 0.037). There were no statistically significant correlations between breastfeeding frequency and infant WLZ at any age or between breastfeeding duration and infant LAZ at any age. There were no statistically significant correlations between breastfeeding frequency and infant LAZ or between breastfeeding duration and infant WLZ at any time point.

**Hypothesis 1a** **(H1a).** *Greater Prenatal Maternal Circadian Cortisol Release Will Be Related to Lower Infant WLZ from Birth to 6 Months.*

A two-way mixed ANOVA was performed to examine the relationship between maternal prenatal AUC_G_ and infant WLZ from birth to 6 months. There was a significant main effect of age (*F*(1, 55) = 10.75, *p* = 0.002, η_p_^2^ = 0.164; See Table 3 for descriptive statistics), such that from birth to 6 months of age, infants’ WLZ increased. The main effect of AUC_G_ and the interaction between age and AUC_G_ were not significant (*p* = 0.242 and *p* = 0.707). Similarly, a two-way ANOVA for maternal prenatal CAR and infant WLZ from birth to 6 months revealed a significant main effect for age (*F*(1, 67) = 44.82, *p* < 0.001, η_p_^2^ = 0.401). The main effect of CAR and the interaction between age and CAR were not statistically significant (*p* = 0.061 and *p* = 0.252).

**Hypothesis 1b** **(H1b).** *Greater Prenatal Maternal Circadian Cortisol Release Will Be Related to Lower Infant LAZ from Birth to 6 Months.*

For maternal prenatal AUC_G_ and infant LAZ from birth to 6 months, a two-way mixed ANOVA revealed a significant, positive main effect of age (*F*(1, 56) = 12.69, *p* = 0.001, η_p_^2^ = 0.185; See Table 2 for descriptive statistics). The main effect of AUC_G_ and the interaction between age and AUC_G_ was not statistically significant (*p* = 0.207 and *p* = 0.573). A two-way mixed ANOVA was performed to examine the relationship between maternal prenatal CAR and infant LAZ from birth to 6 months. There was a significant, positive main effect of age (*F*(1, 68) = 102.55, *p* < 0.001, η_p_^2^ = 0.601), however, the main effect of CAR and the interaction between age and CAR were not statistically significant (*p* = 0.055 and *p* = 0.132).

**Hypothesis 2a** **(H2a).** *The Negative Relationship between Greater Maternal Prenatal Cortisol and Lower Infant WLZ at 6 Months Will Be Exacerbated by Less Breastfeeding at 6 Months*

A moderation model where maternal prenatal AUC_G_ = X, infant WLZ at 6 months = Y, and breastfeeding frequency = W was not found to be statistically significant (*n* = 56, *F*(3, 52) = 0.41, *R*^2^ = 0.02, *p* = 0.749). A similar model where breastfeeding duration = W, X and Y held constant, was not found to be statistically significant (*n* = 60, *F*(3, 56) = 0.33, R^2^ = 0.02, *p* = 0.806). Moderation models where maternal CAR = X, infant WLZ at 6 months = Y, and breastfeeding frequency *(n* = 68, *F*(3, 64) = 1.08, *R*^2^ = 0.05, *p* = 0.366) and duration = W (*n* = 72, *F*(3, 68) = 0.76, *R*^2^ = 0.03, *p* = 0.519) were not found to be statistically significant.

**Hypothesis 2b** **(H2b).** *The Negative Relationship between Greater Maternal Prenatal Cortisol and Lower Infant LAZ at 6 Months Will Be Exacerbated by Less Breastfeeding at 6 Months.*

A moderation model where maternal prenatal AUC_G_ = X, infant LAZ at 6 months = Y, and breastfeeding frequency = W was not statistically significant (*n* = 56, *F*(3, 52) = 1.21, *R*^2^ = 0.07, *p* = 0.314). Similarly, a moderation model where maternal prenatal AUC_G_ = X, infant LAZ at 6 months = Y, and breastfeeding duration = W was not found to be statistically significant (*n* = 60, *F*(3, 56) = 1.08, *R*^2^ = 0.05, *p* = 0.365). Moderation models where maternal CAR = X, infant LAZ at 6 months = Y, and breastfeeding frequency (*n* = 68, *F*(3, 64) = 0.74, *R*^2^ = 0.03, *p* = 0.533) and duration = W (*n* = 72, *F*(3, 68) = 0.49, *R*^2^ = 0.02, *p* = 0.692) were not found to be statistically significant.

## 4. Discussion

The current study proposed to fill a gap in the existing literature on how maternal prenatal cortisol and breastfeeding influence growth outcomes in later infancy, as previous research has primarily focused on growth outcomes at birth [2]. We hypothesized that maternal prenatal cortisol would be negatively correlated with infant growth across infancy, however, we found limited statistically significant associations among maternal prenatal cortisol and infant growth outcomes. We found that infant LAZ at birth and maternal prenatal CAR were negatively associated, which supports that higher levels of maternal prenatal cortisol might result in in utero growth restriction [17,18]. This is consistent with existing literature, which highlights significant relationships between flattened circadian saliva cortisol profiles and lower infant birth weight, length, and head circumference [19]. While it is unclear why these relationships were not seen using WLZ at birth, it could be that because of the significant effect of cortisol on length, controlling for length in reference to weight limited the covariance between cortisol and WLZ. This suggests that birth length may be more affected by prenatal cortisol exposure than birth weight, though additional studies should test this preliminary hypothesis. We also found that there was a positive correlation between infant LAZ at 13–14 months and maternal prenatal CAR. The direction of these relationships supports previous literature that indicates that infants who are smaller for their gestational age have rapid catch-up growth gains [20]. No known empirical literature highlights age differences in relations between in utero cortisol exposure and anthropometry over the first 1.5 years. However, longitudinal studies examining catch-up growth in normative and small-for-gestational-age (SGA) infants have supported that the most rapid rate of catch-up growth may be between birth to 4 months of age and that differences in growth velocity among SGA and normative infants may extend until 12 months [20]. In other words, it may be that the best ages to assess associations between prenatal cortisol and anthropometry are in early (i.e., birth to 4 months) and late (i.e., around 12 months) infancy. The current study finding represents a novel contribution to how early prenatal CAR affects long term growth by examining later time points in infancy. For example, other studies have reported that maternal prenatal HPA axis functioning, including CAR, mediates relations between maternal socioeconomic status and infant birth weight due to direct and indirect effects of glucocorticoids on infant growth and development [21], but these findings have not extended into early childhood. Examining continued rapid growth into infancy and early childhood is important to further our understanding of how this rapid growth is related to outcomes across the lifespan. For instance, previous research suggests that increased risk of Type 2 diabetes and coronary events in adults was associated with low birth weight, suggesting that this rapid growth might have serious implications for health outcomes [22,23].

Although we hypothesized that we would see impacted infant growth from birth to 6 months as a result of early cortisol exposure, we did not find significant main effects. However, we did find that there was a significant effect of age for infant WLZ and LAZ. Given that both age and sex are accounted for in the standardized z-scores used for analyses, this suggests that there are other factors that influence infant growth outcomes in this sample (e.g., mental and physical health and sociodemographic characteristics). However, based upon the recruitment strategy and exclusion criteria in this community sample, there was limited variability in these measures, so they were not examined in relation to current study predictors and outcomes. In addition, an examination of potential covariates that might influence this relationship revealed consistently non-significant results in the study sample, including gravida, gestational age, gestational weight gain, and diet.

Particularly, although our sample contained infants who were born notably smaller than average (mean WLZ was −1.02), we found that at the first postnatal follow-up session, infants were above average (average WLZ was 0.61). This change in growth could be attributed to our relatively low-risk sample. The inclusion and exclusion criteria for the current study excluded most mothers who would be considered high risk (e.g., due to substance use, major medical/psychiatric health diagnosis, or pregnant with multiples). Furthermore, our study sample had relatively high rates of breastfeeding mothers, with 64% of infants being breastfed through 6 months compared to the national average of 58% at this timepoint [24], which may be due to sociodemographic characteristics of the current sample, which place them at a lower risk. Breastfeeding is associated with heavier infants [11,12], thus, we could see a higher average WLZ due to the high rates of breastfed infants in our sample. Given the lack of significant associations between cortisol and infant growth in our sample, we are not able to conclude that this is the same rapid catch-up growth exhibited by infants with low birth weight due to elevated cortisol exposure, however, it is possible that we are seeing rapid catch-up growth due to lower birth weight or that we are capturing unique diet or feeding practices within our sample. It is critical for future studies to disentangle prospective predictors to better elucidate how cortisol exposure and breastfeeding may impact infant growth trajectories.

### 4.1. Practical Implications

The findings in the current study include important implications for infant growth outcomes. We found evidence for a continued association between elevated maternal prenatal cortisol with later infant growth outcomes suggesting that continued catch-up growth may occur in the offspring of mothers with higher cortisol levels during pregnancy. Higher cortisol levels in pregnancy may be the result of dysregulation of the HPA axis secondary to anxiety, depression, posttraumatic stress disorder, schizophrenia [2], excessive maternal weight, or disorders such as Cushing Syndrome. Therefore, screening for these prenatal risk factors and cortisol elevations may help to identify offspring with anthropometric risk factors for poor health across the lifespan. Additionally, the careful longitudinal assessment and tracking of these important risk factors across the first year of life can help to understand the effectiveness of interventions to manage catch-up growth and adiposity in infancy and early childhood. However, more basic research and clinical studies are needed to understand the best way to implement such interventions.

Additionally, the current study sample was drawn from a rural area that is classified as a Health Provider Shortage Area for primary care and mental health services, and although the sample is a relatively low-risk community sample, our participants represent a population with fewer studies describing maternal health and infant outcomes and may have fewer resources available for women and infants. Therefore, additional studies are needed to better understand the unique risk and resiliency factors in these settings in relation to offspring growth.

### 4.2. Study Limitations and Future Directions

Current study findings should be interpreted while considering several limitations. Since the relations between breastfeeding, maternal prenatal cortisol, and infant growth have not been previously examined, it is possible that a relationship between the three variables does not exist, however, it could be that our community sample of low-risk mothers could influence the absence of a relationship among these variables that does, in fact, exist. In order to test this possibility, studies could compare differences between normative and at-risk samples with regard to these important variable relationships. Furthermore, our sample consisted primarily of relatively high and homogeneous socioeconomic status, which could factor into lower levels of cortisol during pregnancy. Lastly, in regard to our sample, there were participants who failed to complete their saliva samples and missing data across time points led to relatively small sample sizes in several analyses, which were likely underpowered with regard to the current study’s analysis plan, particularly when examining moderation effects. It is possible that there were significant and meaningful differences in cortisol concentrations of women who did not complete saliva samples and therefore are not represented in the current data. It is likely that there is a difference between mothers who were more involved or less involved with the study though given the lack of heterogeneity in the current study sample, sociodemographic differences do not appear to exist between mothers with (*M_AGE_* = 26.5 years, largest percentage (27%) with annual income of USD 50,000–75,000, and mostly (68%) completed partial college or BS/BA, (94%) White, and (62%) members of Church of Jesus Christ Latter Day Saints) and without (*M_AGE_* = 27.4 years, largest percentage (21%) with annual income of USD 50,000–75,000, and mostly (79%) completed partial college or BS/BA, (92%) White, and (64%) members of Church of Jesus Christ Latter-Day Saints) prenatal cortisol samples. Beyond the current sample, variations in the formulas used to calculate cortisol (i.e., differences in AUC formulas) may influence findings across research labs and contribute to inconsistencies in findings [14]. In future studies, the inclusion of a larger, more diverse sample would strengthen the confidence of findings related to relations between breastfeeding, maternal cortisol, and infant growth. Additionally, intentional recruitment of women with elevated cortisol, or a higher risk sample, could provide a clear and more accurate representation of variation in cortisol levels across pregnant women and elucidate potential existing relationships not captured in the present study. Similarly, more diverse samples with regard to mental health, health behaviors, and sociodemographic characteristics would allow researchers to investigate these factors as covariates and better understand relationships among prenatal cortisol, breastfeeding, and infant growth.

## 5. Conclusions

Taken together, current study results suggest that in utero exposure to cortisol negatively predicts infants’ size at birth, but positively predicts similar anthropometric indices at 13–14 months of age. This may be due to the effects of cortisol on fetal growth restriction, which may result in infants who are born earlier and/or smaller and then engage in rapid catch-up growth. This converges with current study findings that there were significant increases in WLZ and LAZ from below average to above average from birth to 6 months of age. The current study also found significant negative associations between breastfeeding and 10-month anthropometric outcomes, which may be due to the positive effects of breastfeeding for managing a healthy weight in infants. Specifically, breastfeeding has positive benefits for infant nutrition, immune functioning and emotional development compared to formula feeding [25]. In fact, there may be an overuse of nutrient-dense formula or non-breastmilk foods to accelerate infant weight and length trajectories in developing and developed countries, which may put offspring at risk for poorer immune functioning and several diseases, including obesity, across the lifespan [25]. Although the current study does not examine breastfeeding duration and frequency with regard to exclusivity or measure specific amounts of breast milk ingested, the two variables serve as a proxy for the relative amount of breastfeeding that infants receive. Follow-up work should consider multiple risk and resiliency factors that may explain these notable shifts in standardized anthropometry and if possible, multi-method assessment to more comprehensively quantify breastfeeding amount. Additionally, current study findings should be interpreted within the scope of existing study limitations and future studies should examine relations among psychophysiological stress, breastfeeding behaviors, and infant development in larger, more heterogenous samples with unique risk factors.

## Figures and Tables

**Table 1:** Sample Demographics.

Anthropometric Variable	Mean/Standard Deviation
Age	27/4
Race/Ethnicity	N/%
White/Caucasian	116/92.8
Black/African American	2/1.6
Native Hawaiian or other Pacific Islander	2/1.6
American Indian/Alaska Native	3/2.4
Hispanic/Latino	16/12.8
Asian	1/0.8
Other	8/6.4
Marital Status	N/%
Single/never married	10/8%
Married	99/79.2
Divorced	3/2.4
Committed relationship	9/7.2
Engaged	4/3.2
Religious Affiliation	N/%
Agnostic	3/3.1
Assembly of God	2/2.1
Atheist	2/2.1
Baptist	2/2.1
Catholic	5/5.2
Lutheran	2/2.1
Methodist	1/1
Church of Jesus Christ of Latter-Day Saints	60/62.5
Non-denominational	10/10.4
Pentecostal	1/1
Presbyterian	1/1
Other	12/12.5
Prefer not to say	9/9.4
Income	N/%
<$5000	2/1.6
$5000–9999	3/2.4
$10,000–19,999	19/15.2
$20,000–29,999	24/19.2
$30,000–39,999	15/12
$40,000–49,999	12/9.6
$50,000–74,999	31/24.8
$75,000–99,999	9/7.2
>/=$100,000	10/8
Education	N/%
Junior high school	1/0.8
Partial high school	4/3.2
High school	18/14.4
Partial college	44/35.2
Standard college or university	46/36.8
Graduate training with a degree	12/9.6

**Table 2:** Correlations among Maternal Prenatal Diurnal Cortisol Variables, Breastfeeding, and Infant Standardized Anthropometric Variables from Birth to 18 Months Postpartum.

Anthropometric Variable	Correlation Results	AUCg	Average CAR	Breastfeeding Duration	Breastfeeding Frequency
**WLZ (Birth)**	Pearson Correlation	0.118	0.213	0.144	0.036
	Sig. (2-tailed)	0.383	0.079	0.176	0.740
	*N*	57	69	90	85
**LAZ (Birth)**	Pearson Correlation	−0.153	* −0.247	0.004	−0.038
	Sig. (2-tailed)	0.251	0.039	0.971	0.729
	*N*	58	70	91	86
**WLZ (6 months)**	Pearson Correlation	−0.087	0.086	−0.053	−0.195
	Sig. (2-tailed)	0.509	0.475	0.612	0.067
	*N*	60	72	95	89
**LAZ (6 months)**	Pearson Correlation	−0.135	−0.123	−0.036	−0.126
	Sig. (2-tailed)	0.305	0.303	0.726	0.240
	*N*	60	72	95	89
**WLZ (10 months)**	Pearson Correlation	−0.207	−0.086	* −0.344	−0.316
	Sig. (2-tailed)	0.396	0.695	0.037	0.064
	*N*	19	23	37	35
**LAZ (10 months)**	Pearson Correlation	0.237	0.173	−0.276	** −0.468
	Sig. (2-tailed)	0.328	0.431	0.099	0.005
	*N*	19	23	37	35
**WLZ (13–14 months)**	Pearson Correlation	−0.135	−0.312	−0.142	−0.194
	Sig. (2-tailed)	0.501	0.083	0.357	0.219
	*N*	27	32	44	42
**LAZ (13–14 months)**	Pearson Correlation	−0.069	* 0.378	−0.057	−0.224
	Sig. (2-tailed)	0.733	0.033	0.713	0.154
	*N*	27	32	44	42
**WLZ (18 months)**	Pearson Correlation	0.049	0.165	−0.200	−0.178
	Sig. (2-tailed)	0.813	0.367	0.199	0.265
	*N*	26	32	43	41
**LAZ (18 months)**	Pearson Correlation	−0.200	−0.077	0.081	0.062
	Sig. (2-tailed)	0.328	0.675	0.607	0.700
	*N*	26	32	43	41

Note: WLZ = sex and age adjusted weight-for-length z-score, LAZ = sex and age adjusted length-for-age z-score, CAR = cortisol awakening response and AUC_G_ = cortisol area under the curve with respect to ground. * *p* < 0.05, ** p < 0.01.

**Table 3:** Infant Anthropometric Variable Descriptive Statistics.

Infant Anthropometric Variable	Mean (Standard Deviation)
Infant Birth Weight (kg)	3.39 (0.47)
Infant Birth Length (cm)	51.57 (3.02)
Birth Weight-for-Length Z-Score	−1.02 (1.82)
Birth Length-for-Age Z Score	1.08 (1.58)
Infant 6-Month Weight (kg)	7.59 (0.83)
Infant 6-Month Length (cm)	65.03 (2.35)
6-Month Weight-for-Length Z-Score	0.61 (0.98)
6-Month Length-for-Age Z-Score	−0.86 (1.06)
